# Natural Product Repertoire of the Genus *Amphimedon*

**DOI:** 10.3390/md17010019

**Published:** 2018-12-30

**Authors:** Nourhan Hisham Shady, Mostafa A. Fouad, Mohamed Salah Kamel, Tanja Schirmeister, Usama Ramadan Abdelmohsen

**Affiliations:** 1 Department of Pharmacognosy, Faculty of Pharmacy, Deraya University, Universities Zone, P.O. Box 61111 New Minia City, 61519 Minia, Egypt; noura_shady2013@yahoo.com (N.H.S.); mskamel@yahoo.com (M.S.K.); 2 Department of Pharmacognosy, Faculty of Pharmacy, Minia University, 61519 Minia, Egypt; m_fouad2000@yahoo.com; 3 Institute of Pharmacy and Biochemistry, Johannes Gutenberg University, Staudingerweg 5, 55128 Mainz, Germany

**Keywords:** marine natural products, sponges, *Amphimedon*, alkaloids, ceramides, bacteria

## Abstract

Marine sponges are a very attractive and rich source in the production of novel bioactive compounds. The sponges exhibit a wide range of pharmacological activities. The genus *Amphimedon* consists of various species, such as *viridis*, *compressa*, *complanata*, and *terpenensis*, along with a handful of undescribed species. The *Amphimedon* genus is a rich source of secondary metabolites containing diverse chemical classes, including alkaloids, ceramides, cerebrososides, and terpenes, with various valuable biological activities. This review covers the literature from January 1983 until January 2018 and provides a complete survey of all the compounds isolated from the genus *Amphimedon* and the associated microbiota, along with their corresponding biological activities, whenever applicable.

## 1. Introduction

The broadness in the discovery of natural marine products have increased over the last two decades with the number of new compounds discovered per year similarly increasing [1]. One of the richest sources of marine natural products, among all the marine organisms investigated in literature, are the marine sponges. Nearly thirty percent of all marine natural products discovered in history come from marine sponges [2,3,4]. Sponges have been considered an important source of new bioactive natural products [5], although the role of microbial symbionts as the producers of sponge metabolites needs more further research in the future. The number of metabolites reported from sponges varies from year to year, e.g., in 2014, 283 new structures were described from the phylum Porifera. By 2015, the number of metabolites (291) remained similar to 2014 and constant in comparison to previous years [6]. In 2016, 224 new compounds were reported from sponges. This reduction may represent the focus changing from studying sponges to studying microorganisms. However, sponges remain one of the important groups in the discovery of new bioactive compounds [7]. Wide ranges of microbes are associated with sponges, these include heterotrophic bacteria, cyanobacteria, dinoflagellates, and diatoms [8]. These microbes all play several roles in the sponges and affect sponge health, growth rates, and the ability to defend against predators [9]. The interaction between sponges and the associated microorganism could help in the nutrition of the sponge [10], or in the fixation of nitrogen [11]. The most important role, however, is the production of secondary metabolites as antifungals and antibiotics [12]. The genus *Amphimedon* (Kingdom: Animalia; phylum: Porifera; class: Demospongiae; sub class: Heteroscleromorpha; order: Haplosclerida; family: Niphatidae) is reported to be a very rich source of bioactive metabolites.

Various chemical and biological investigations, particularly for the different extracts of the undescribed species of the genus *Amphimedon*, can be found in previous literature. These investigations confirmed that the genus, *Amphimedon*, is rich in different classes of natural products such as alkaloids, and rich in different subclasses such as manzamine alkaloids [13,14], purine-based alkaloids [15], pyridine-based alkaloids [16], 3-alkylpyridine glycosides [17], and macrocyclic lactones/lactams [18]. In addition, ceramides and cerebrosoides were isolated from the marine sponges of the genus *Amphimedon* [19]. Moreover, fatty acids were also reported from *Amphimedon* sp. [20].

The marine sponge *Amphimedon* sp. has been collected from different regions, including Okinawa and Fukuoka in Japan [19,21]. Biological investigations of the alkaloids and fatty acids isolated from *Amphimedon* have shown that some of these compounds possess antimicrobial [22], antitrypanosomal [23], and anticancer [24] properties. In addition, discovery of the natural metabolites from the four sponges *viridis*, *compressa*, *complanata*, and *terpenensis* were reported in previous literature. In this review, we present an overview on the chemical structures of the marine metabolites from the marine sponges of the genus *Amphimedon*, their associated microorganisms, biological activities, and their places of collection whenever applicable.

## 2. *Amphimedon compressa*

**Figure 1:**
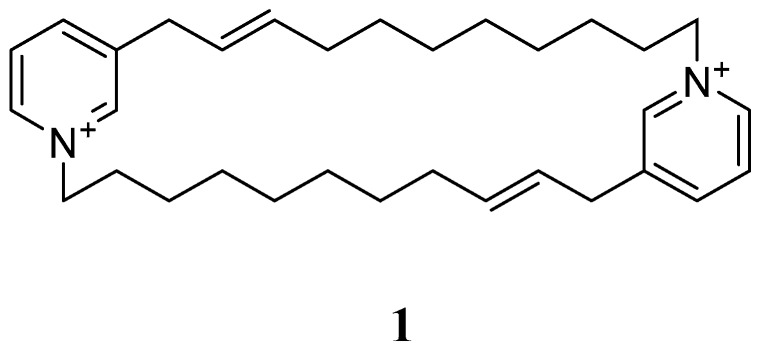
The chemical structure of 8,8′-dienecyclostellettamine (**1**).

Cyclostellettamine [25] is considered an important precursor of the manzamine alkaloids. Its related compound 8,8′-dienecyclostellettamine **1** is an alkyl pyridine alkaloid, as shown in Figure 1, and was isolated from the marine sponge *Amphimedon compressa*, which was collected in Florida.

Compound **1** shows potent antibacterial and antifungal activities against six of the seven tested microorganisms, *Candida albicans*, *Escherichia coli*, *Pseudomonas aeruginosa*, *Cryptococcus neoformans*, Methicillin-resistance *staphylococcus aureus*, and *Aspergillus fumigatus* with IC_50_ values of 0.4, 1.3, 2.1, 2.5, 0.25, and 0.3 mg mL^−1^, respectively.

**Figure 2:**
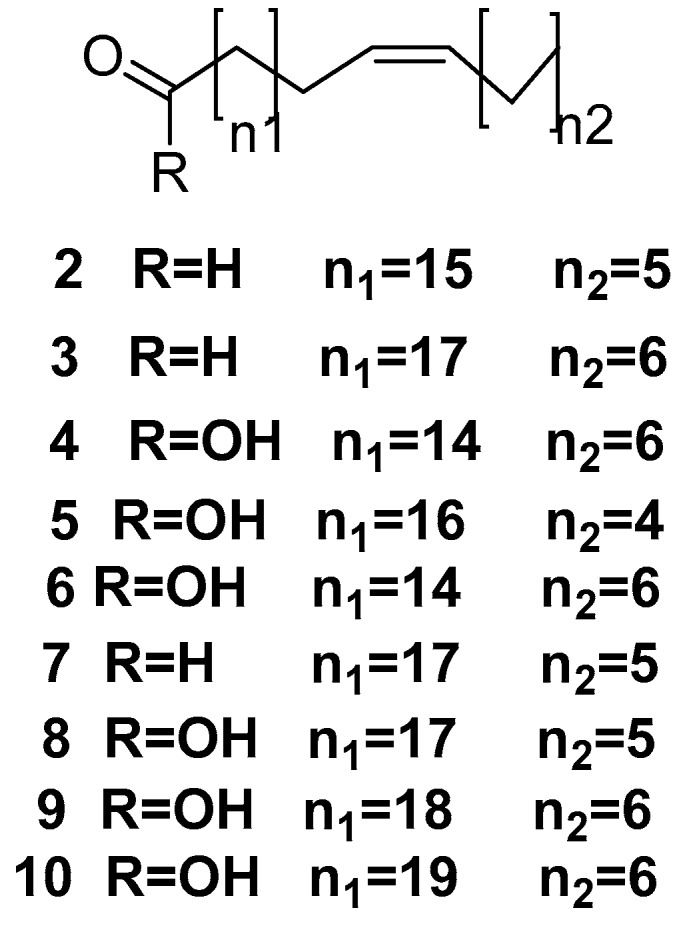
The chemical structures of the compounds (**2**–**10**).

Amphitoxin [26] was isolated from different marine sponges belonging to the order Haplosclerida, which is found in relatively high quantities in extracts of *Amphimedon compressa*, collected near the coast of San Salvador Island. Amphitoxin is a polymeric compound containing 3-alkyl- and 3-alkenyl pyridinium units and contains a mixture of high-molecular-weight pyridinium salts. It is similar in structure to a polymeric pyridinium alkaloid, which has recently been isolated from a sponge belonging to the genus *Callyspongia* (*C. fibrosa*). However, the amphitoxin of *Amphimedon compressa* lacks the carbon–carbon double bond in the side-chain and its molecular weight has not yet been established precisely. The amphitoxin showed ichthyotoxic activity towards *Xiphophorus variatus* (moon fish) and insecticidal activity against *Cylas formicarius elegantulus* (sweet potato weevil) in concentrations of 0.2, 0.8, and 0.4 ppm and corresponding mortality rates of 30, 20 and 10%, respectively, with a life span of 72 h. The toxicity towards the moon fish was evidenced by the high mortality rates. In amphitoxin concentrations of 1000 and 100 ppm, the lifespan of the moon fish is <30 min and <60 min, respectively, thus demonstrating a mortality rate of 100%. [27].

(Z)-17-tricosenal **2**, 19-hexacosenal **3**, 16-tricosenoic acid **4**, 18-tricosenoic acid **5**, 16-tricosenoic acid **6**, 19-pentacosenal **7**, 19-pentacosenoic acid **8**, 20-hepacosenoic acid **9**, and 21-octacosenoic acid **10**, as shown in Figure 2, are mono-unsaturated phospholipid fatty acids isolated from *Amphimedon compressa* [28].

**Figure 3:**
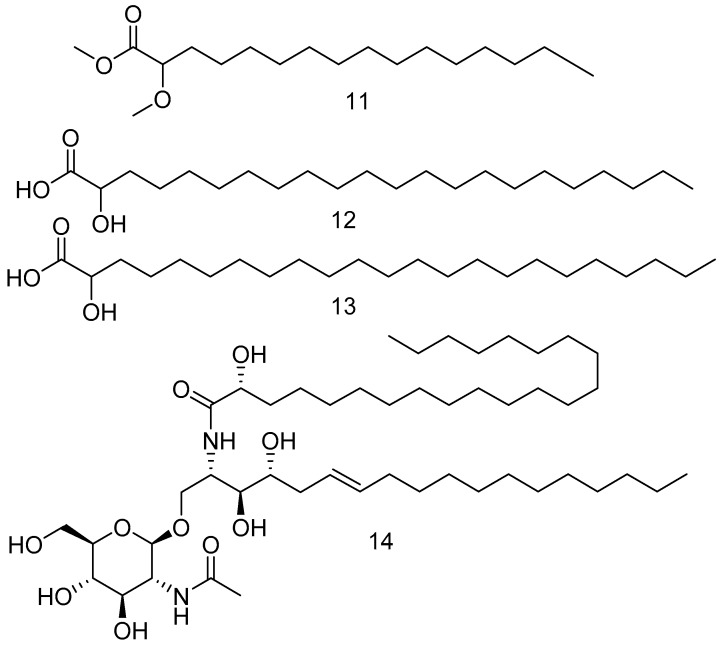
The chemical structures of the compounds (**12**–**14**).

In addition to the previously isolated fatty acids, methyl 2-methoxyhexadecanoate **11** (Figure 3), 2-hydroxydocosanoic acid **12** and 2-hydroxytricosanoic acid **13** [29] have been isolated from *Amphimedon compressa* [30].

Acetamidoglucosyl ceramide **14**, as shown in Figure 3, was isolated from *Amphimedon compressa* collected from the coast of Key Largo, Florida [31].

## 3. *Amphimedon viridis*

**Figure 4:**
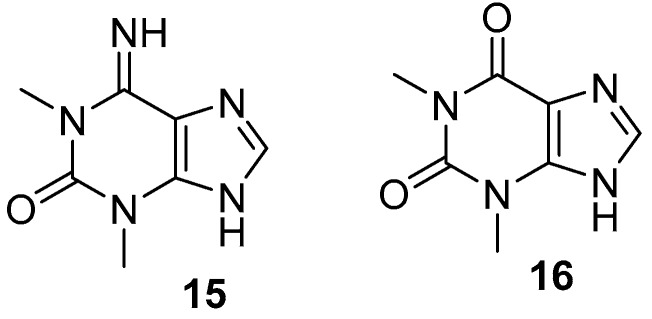
The chemical structures of the compounds (**15**, **16**).

1,3-Dimethylisoguanine **15** [32] is a purine-based alkaloid, as shown in Figure 4, which was isolated from *Amphimedon viridis*. The alkaloid induces contractions when obtained by a transmural electrical stimulation in a guinea pigs’ longitudinal muscle/myenteric plexus in a dose-dependent manner [33].

**Figure 5:**
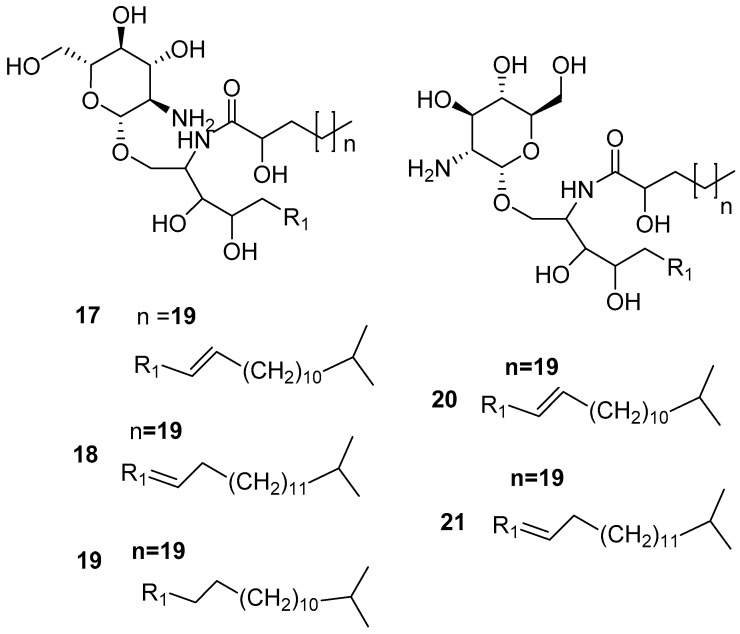
The chemical structures of the compounds (**17**–**21**).

Theophylline **16** [32] is another purine-based alkaloid isolated from *Amphimedon viridis*, which was previously isolated from the plant source *Camellia sinensis* [34]. Furthermore, cerebrosoides amphicerebroside B-F **17**–**21**, as shown in Figure 5, [35] were obtained from this sponge and were collected from the Red Sea.

## 4. *Amphimedon complanata*

**Figure 6:**
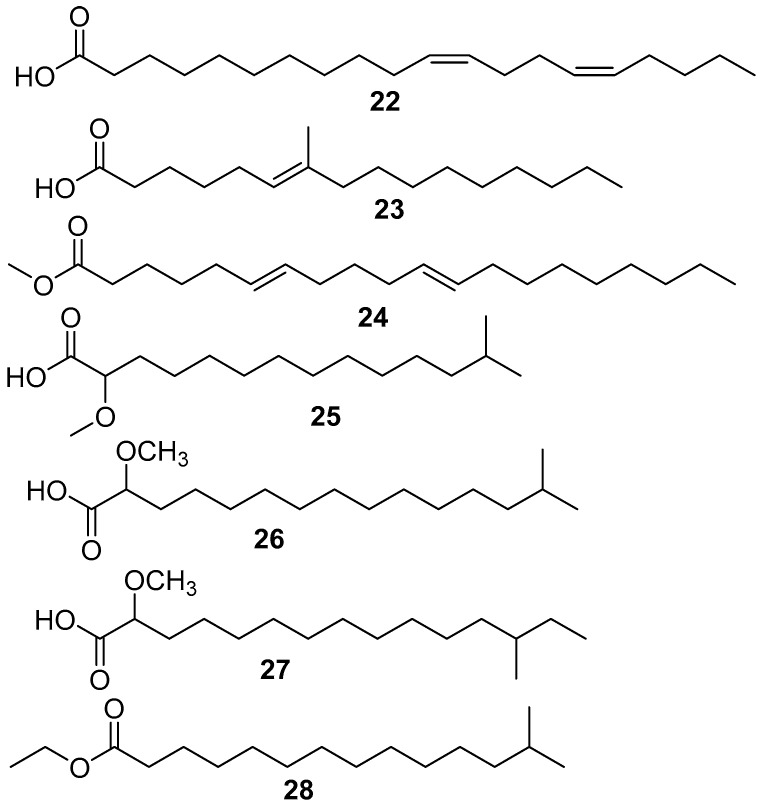
The chemical structures of the compounds (**22**–**28**).

*Amphimedon complanata* is a species of sponge rich in fatty acids, such as 11,l5-icosadienoic acid **22**, 7-methyl-6-hexadecenoic acid **23**, and 6,11-icosadienoic acid **24** [36], and was isolated from a sponge collected near the shelf edge of La Parguera at a depth of 80 ft, as shown in Figure 6.

For the first time in nature 2-methoxy-13-methyltetradecanoic acid **25**, 2-methoxy-14-methylpentadecanoic acid **26**, 2-methoxy-13-methylpentadecanoic acid **27**, and ethyl 2-methoxy-13-methyltetradecanoate **28** were identified and isolated from the marine sponge *Amphimedon complanata*, collected in Puerto Rico, as shown in Figure 6 [37].

## 5. *Amphimedon terpenensis*

**Figure 7:**
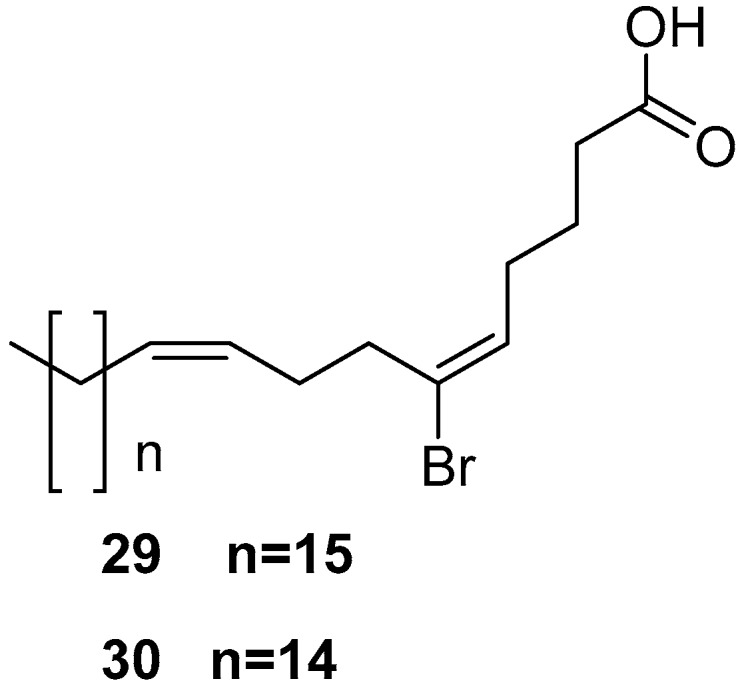
The chemical structures of the compounds (**29**, **30**).

6-Bromo-5*E*,9*Z*-pentacosadienoic acid **29** and 6-bromo-5*E*,9*Z*-tetracosadienoic acid **30**, as shown in Figure 7, are brominated acids and were isolated from *Amphimedon terpenensis*.

## 6. Undescribed Marine Sponges of the Genus *Amphimedon*

One of the classes repeatedly isolated from *Amphimedon* sp. is the manzamine alkaloids. In 1986, Higa and coworkers isolated the manzamine A prototype for this group, a novel cytotoxic β-carboline alkaloid from marine sponge *Haliclona* sp. collected from Manzamo, Okinawa [38]. The isolation of manzamines (B–D), from the marine sponge *Haliclona* sp. subsequently followed [39], as well as ircinals (A–B) from an *ircinia* sponge [40]. In the last two decades more than 80 β -carboline-containing manzamine alkaloids and manzamine-related alkaloids have been isolated from marine sponges following manzamine A discovery [41]. Manzamines are very attractive bioactive natural metabolites due to their wide range of biological applications as well as insecticidal [42], cytotoxic [43], and anti-inflammatory properties [44].

**Figure 8:**
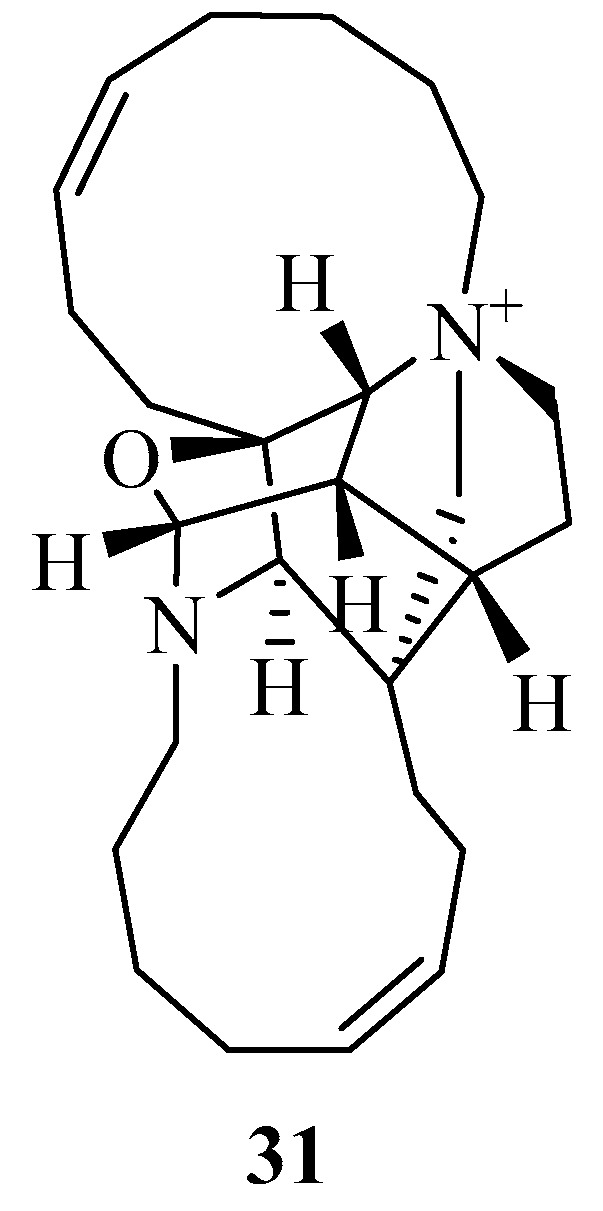
The chemical structure of the compound (**31**).

The first example is zamamiphidin A **31** (Figure 8) [13] which was obtained from the *Amphimedon* sp. collected at Zamami, Okinawa. The compound exhibited antibacterial activity against *Staphylococcus aureus* (MIC, 32 μg/mL) but did not show any activity against *Escherichia coli*, *Bacillus subtilis*, or *Micrococcus luteus* (MIC, >32 μg/mL). The compound also displayed antifungal activities against *Aspergillus niger*, *Trichophyton mentagrophytes*, *Candida albicans*, and *Cryptococcus neoformans* (IC_50_ > 32 μg/mL). The compound did not show cytotoxicity against L1210 murine leukemia or KB human epidermoid carcinoma cells (IC_50_ > 10 μg/mL).

**Figure 9:**
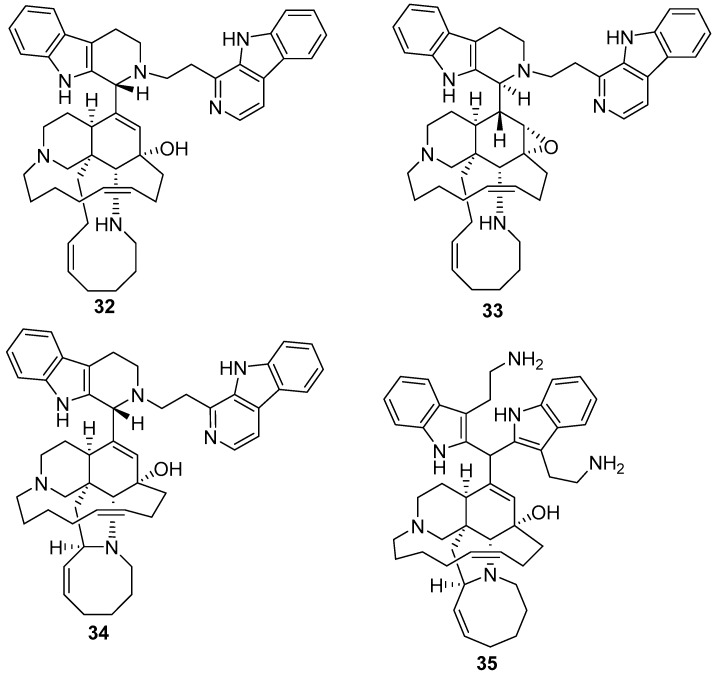
The chemical structures of the compounds (**32**–**35**).

Zamamidine A-C **32**–**34** (Figure 9) [45,46] are other manzamine alkaloids with inhibitory activities against *Trypanosoma brucei brucei* (IC_50_ values of 1.04 mg/mL, 1.05 mg/mL, and 0.27 mg/mL, respectively) and against *Plasmodium falciparum* with IC_50_ values of 7.16 mg/mL, 12.20 mg/mL, 0.58 mg/mL, respectively. Zamamidine D **35** [22], as shown in Figure 9, is the first manzamine alkaloid possessing a 2,2′-methylene bis-tryptamine unit as the aromatic moiety instead of a β-carboline unit. It showed antibacterial activity against *Escherichia coli*, *Stapylococcus aureus*, *Bacillus subtilis*, and *Micrococcus luteus* with MIC values of 32, 8, 8, and 8 μg/μL, respectively. In addition, it showed antifungal activity against *Aspergillus niger*, *Trichophyton mentagrophytes*, *Candida albicans* and *Cryptococcus neoformans* with IC_50_ values of 16, 8, 16, and 2 μg/μL, respectively.

**Figure 10:**
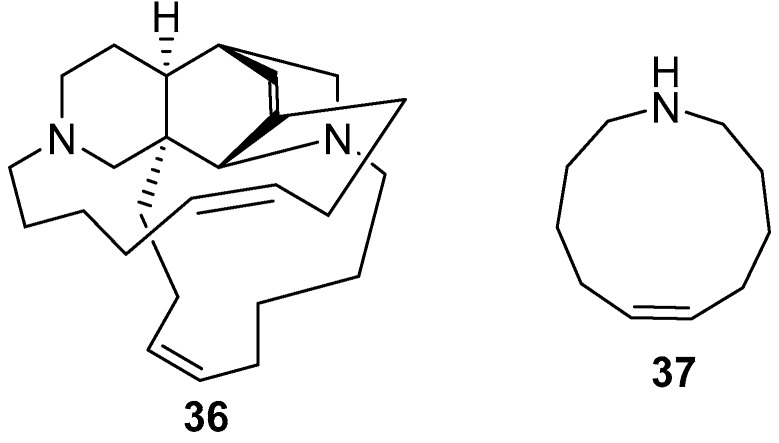
The chemical structures of the compounds (**36**, **37**).

Keramaphidin B **36** and keramaphidin C **37** [21,47,48], as shown in Figure 10, were isolated from *Amphimedon* sp. which was collected from the Kerama Islands, Okinawa.

**Figure 11:**
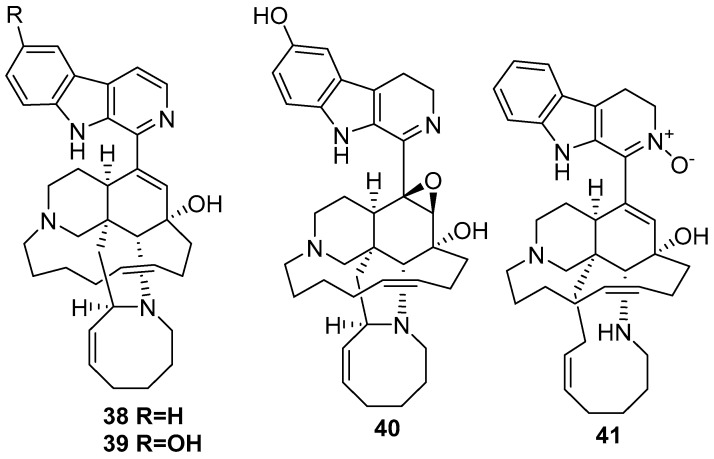
The chemical structures of the compounds (**38**–**41**).

Among the manzamine derivatives 3,4-dihydromanzamine A **38**, 6-hydroxymanzamine A **39** [43], 3,4 dihydro-6 hydroxy-10,11-epoxymanzamine A **40**, and 3,4-dihydromanzamine J *N*-oxide **41** [46], as shown in Figure 11, the 3,4-dihydromanzamine J *N*-oxide **41** compound showed anti-parasitic activity against *T. b. brucei* with an IC_50_ value of 4.44 (μg/mL) and against *P. falciparum* with an IC_50_ value of 7.02 (μg/mL) [23].

**Figure 12:**
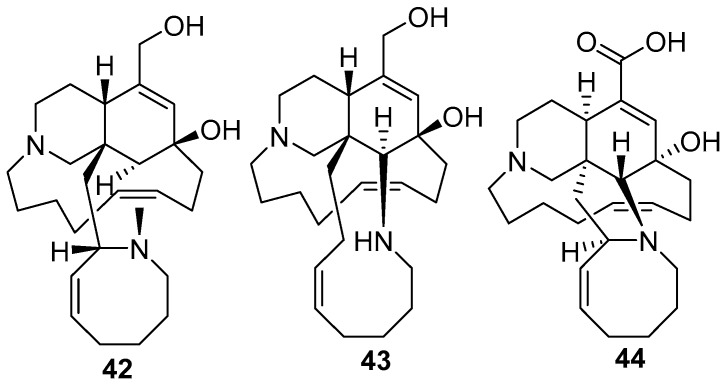
The chemical structures of the compounds (**42**–**44**).

Ircinol A **42**, ircinol B **43** [14], and ircinic acid A **44** (which is the 1-*O*-methyl carboxylic acid analog of ircinal A) [13], as shown in Figure 12, were isolated from *Amphimedon* sp. collected from the Kerama Islands, Okinawa.

Keramamine C **45** [47], manzamine H **46**, and manzamine L **47** displayed cytotoxicity against murine lymphoma LI210 cells and human epidermoid carcinoma KB cells with IC_50_ values of 3.7 and 11.8l µg/mL, respectively. Antibacterial activity against the bacteria *Sarcina lutea*, *Staphylococcus aureus*, *Bacillus subtilis*, and *Mycobacterium* 607 with MICs values of 10, 10, 10, and 5 µg/mL, respectively [21].

**Figure 13:**
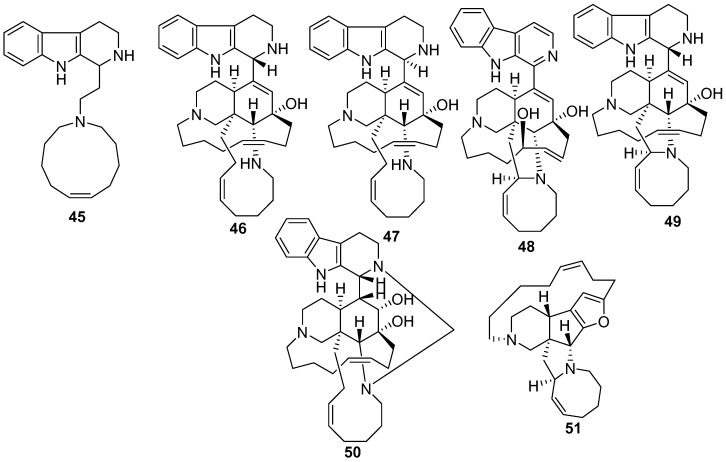
The chemical structures of the compounds (**45**–**51**).

New manzamine-related tetrahydro-carboline alkaloids with a methylene carbon bridge between N-2 and N-27 such as manzamine M **48** [49], manzamine D **49** [21], ma’eganedin A **50**, and nakadomarin A **51** are novel cytotoxic alkaloids from *Amphimedon* sp. collected in Okinawa. Nakadomarin A **51** showed cytotoxicity against murine lymphoma L1210 cells (IC_50_ 1.3 µg/ mL) and exhibited activity against cyclin dependent kinase 4 (IC_50_ 9.9 µg/mL) [50], as shown in Figure 13.

**Figure 14:**
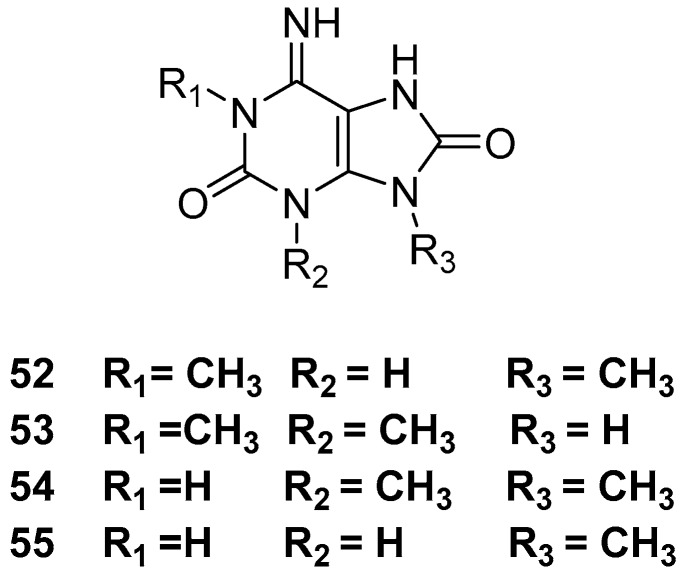
The chemical structures of the compounds (**52**–**55**).

Another compound class isolated from *Amphimedon* sp. are purine-based alkaloids such as 6-imino-1,9-dimethyl-7,9-dihydro-1*H*-purine-2,8(3*H*,6*H*)-dione **52**, 6-imino-1,3-dimethyl-7,9-dihydro-1*H*-purine-2,8(3*H*,6*H*)-dione **53**, 6-imino-3,9-dimethyl-7,9-dihydro-1*H*-purine-2,8(3*H*,6*H*)-dione **54**, and 6-imino-9-methyl-7,9-dihydro-1*H*-purine-2,8(3*H*,6*H*)-dione **55** [15], as shown in Figure 14. The compounds showed neuropharmacological activities with CD_50_ values of 2.4, 54, 18, 18 nmol, respectively, in mouse via the modulation of inhibitory transmission actions in the mammalian CNS.

**Figure 15:**
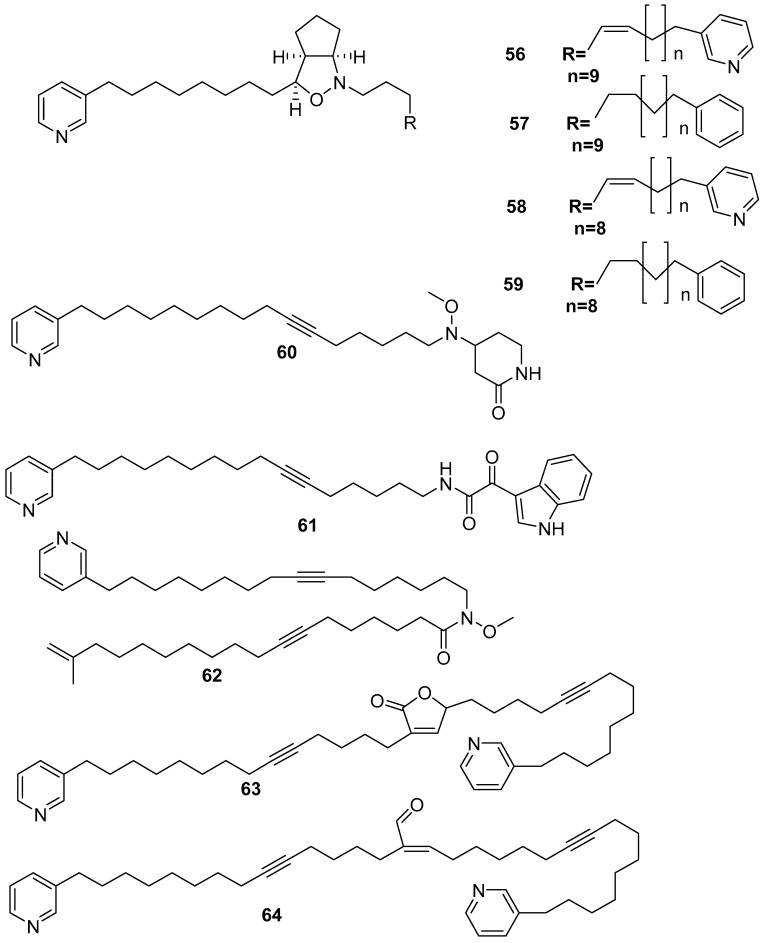
The chemical structures of the compounds (**56**–**64**).

Pyridine-based alkaloid compounds were isolated with high yield from *Amphimedon* sp. An example is pyrinodemin A **56** [51], which showed potent cytotoxicity against the murine leukemia LI210 with an IC_50_ value of 0.058 µg/mL and against KB epidermoid carcinoma cells with an IC_50_ value of 0.5 µg/mL. The related compounds pyrinodemin B–D **57**–**59** [16] exhibited potent cytotoxicity against murine leukemia with IC_50_ values of 0.07, 0.06, and 0.08 µg/mL, respectively. The analogs pyrinodemin E, F **60**, **61** [52], pyrinodemin G, H **62**, and **63** [53] exhibited cytotoxicity against P388 and L1210 murine leukemia cells with IC_50_ values of 5.7 and 8.8 µg/mL, and 9.6 and 2.5 µg/mL, respectively, in vitro. Finally, pyrinodemin I **64** was also isolated from *Amphimedon* sp., as shown in Figure 15.

**Figure 16:**
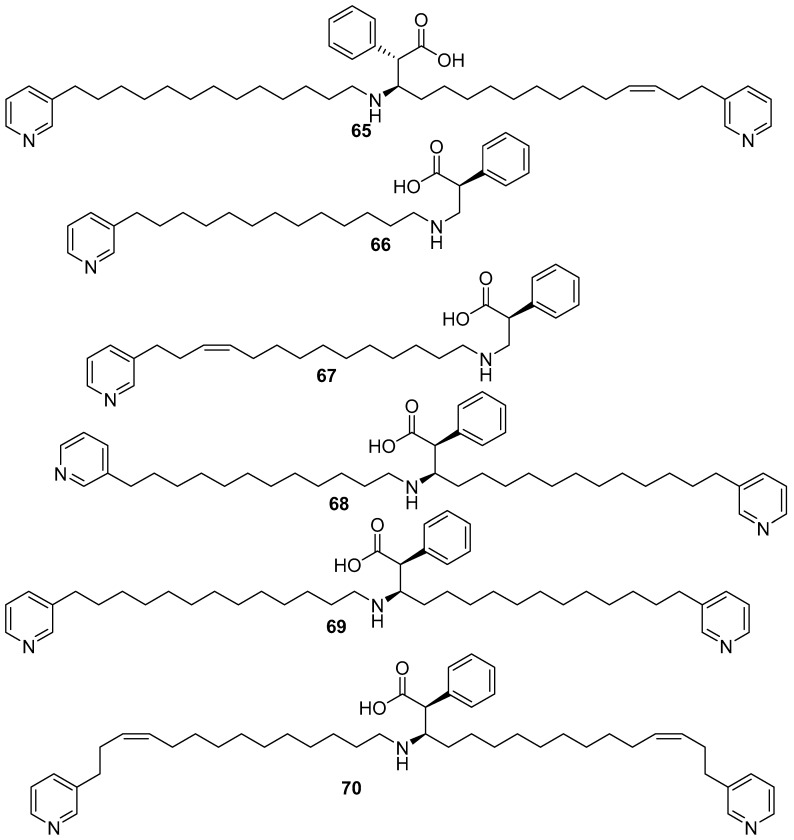
The chemical structures of the compounds (**65**–**70**).

Nakinadine A **65** [54] which was isolated from a sponge collected in Nakijin, Okinawa, is an example of a bis-3-alkyl pyridine alkaloid with a β-amino acid moiety. The compound showed cytotoxicity against L1210 murine leukemia (IC_50_ 1.3 µg/mL) and KB human epidermoid carcinoma cells (IC_50_ 2.5 µg/mL) in-vitro, as shown in Figure 16. The related nakinadine B–F **66**–**70** [55], were also isolated from a sponge collected in Nakijin, Okinawa. Nakinadine B **66** and nakinadine C **67** were shown to exhibit cytotoxicity against L1210 murine leukemia with IC_50_ values of 3.0 and 5.0 µg/mL, respectively, and against KB human epidermoid carcinoma cells with IC_50_ values of 7.0 and >10 µg/mL, respectively, in-vitro, while nakinadines D–F **68**–**70** did not show these activities (IC_50_ > 10 µg /mL).

**Figure 17:**
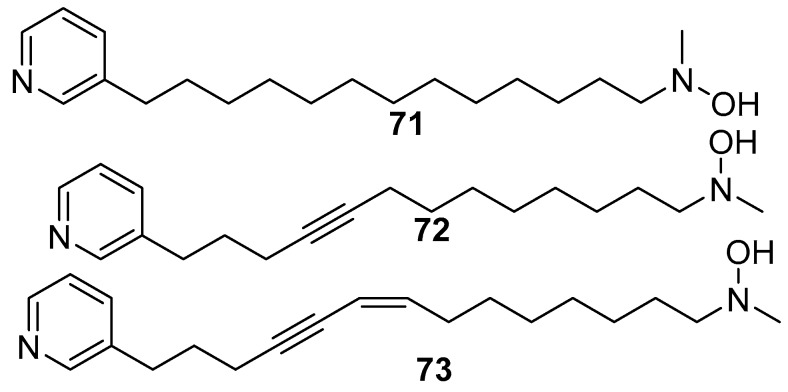
The chemical structures of the compounds (**71**–**73**).

**Figure 18:**
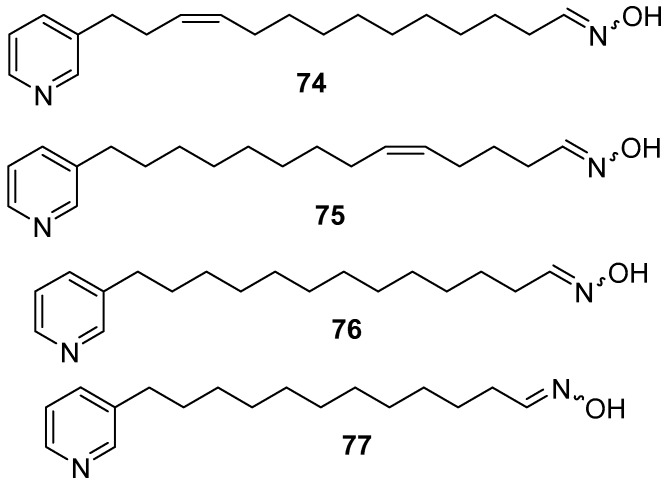
The chemical structures of the compounds (**74**–**77**).

Hachijodine E–G **71**–**73** [24] are also 3-alkylpyridine alkaloids isolated from *Amphimedon* sp. collected off the coast of Hachijo-jima Island. They also showed cytotoxic activity against P388 murine leukemia cells with IC_50_ values of 2.3, 1.0, 1.0 µg/mL, respectively, as shown in Figure 17.

(1*E*,11*Z*)-*N*-Hydroxy-14-(pyridin-3-yl) tetradec-11-en-1-imine **74**, (1*E*,5*Z*)-*N*-hydroxy-14-(pyridin-3-yl) tetradec-5-en-1-imine **75**, (1*E*)-*N*-hydroxy-13-(pyridin-3-yl) tridecan-1-imine **76**, and (1*E*)-*N*-hydroxy-12-(pyridin-3-yl) dodecan-1-imine **77** [16], as shown in Figure 18, are compounds which possess antifungal and antimicrobial activities.

**Figure 19:**
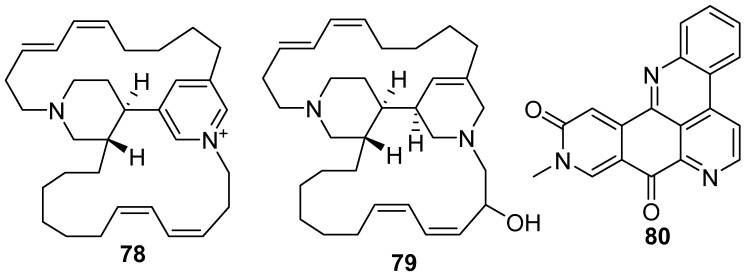
The chemical structures of the compounds (**78**–**80**).

Tetrahydrohalicyclamine A **78** and 22-hydroxyhalicyclamine A **79** (Figure 19) [56], are 3-alkylpiperidine alkaloids isolated from the *Amphimedon* sp. in Southern Japan. These compounds displayed cytotoxic activity inhibiting the growth of P388 cells with IC_50_ values of 2.2 and 0.45 µg/mL, respectively. Amphimedine **80** [57] is a pentacyclic aromatic alkaloid isolated from the *Amphimedon* sp.

**Figure 20:**
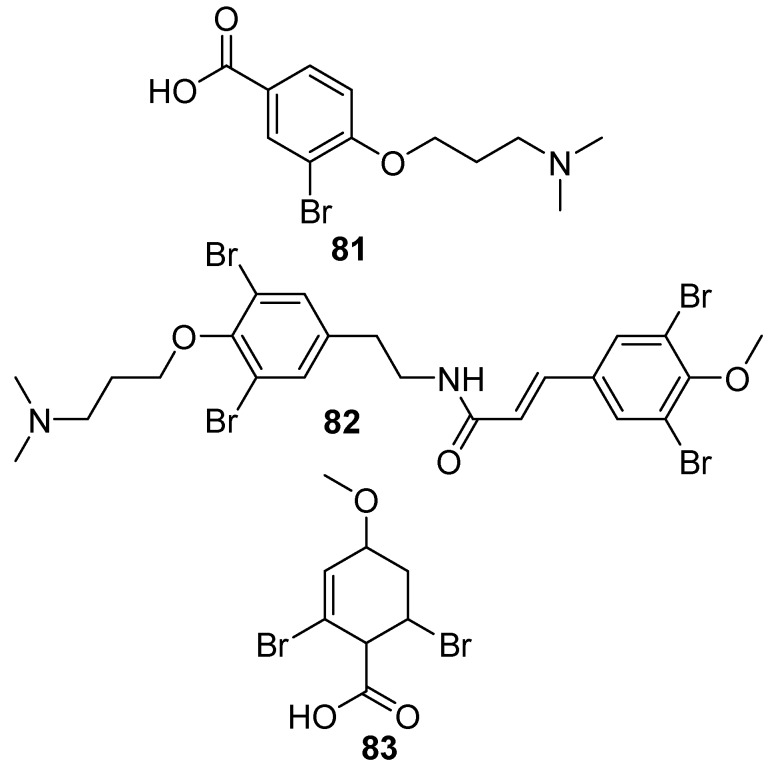
The chemical structures of the compounds (**81**–**83**).

A group of brominated alkaloids, namely amphimedonoic acid **81**, psammaplysene E **82**, and 3,5-dibromo-4-methoxybenzoic acid **83** were isolated from *Amphimedon* sp. collected from the Mitsio Islands, Madagascar, as shown in Figure 20 [58].

**Figure 21:**
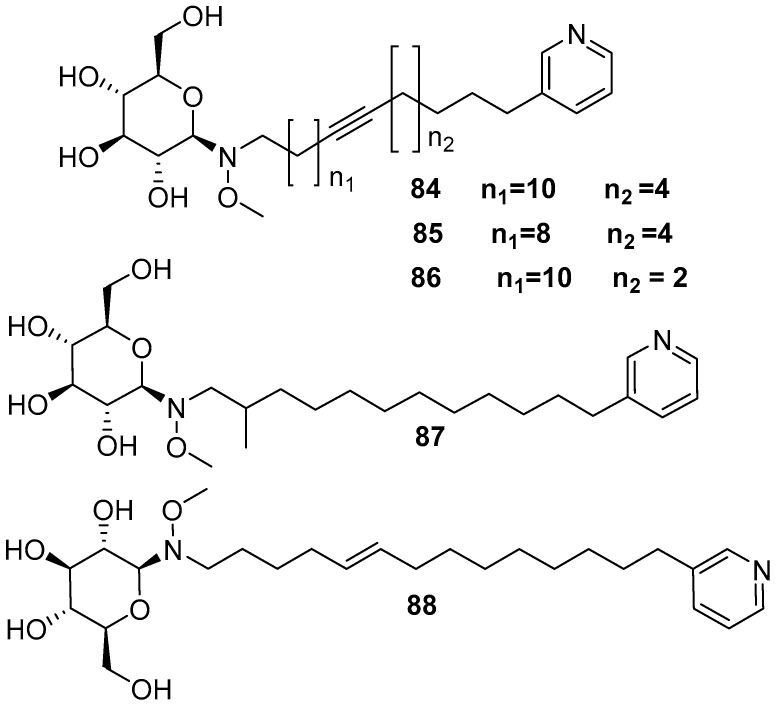
The chemical structures of the compounds (**84**–**88**).

**Figure 22:**
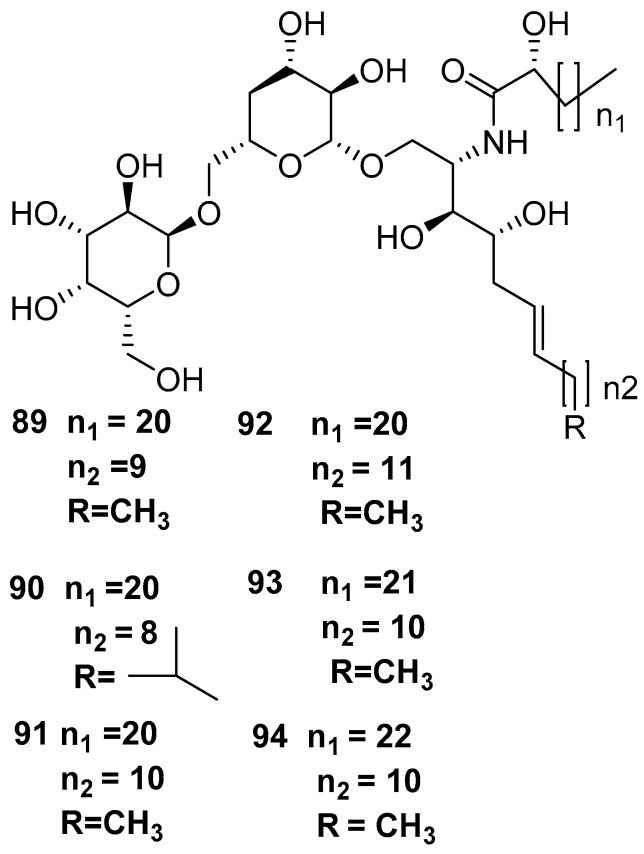
The chemical structures of the compounds (**89**–**94**).

Amphimedoside A **84**, amphimedoside B **85**, amphimedoside C **86**, amphimedoside D **87**, and amphimedoside E **88** [17] are 3-alkylpyridine glycosides isolated from the *Amphimedon* sp. collected from the Hachijo-jima Island, as shown in Figure 21. Amphimelibioside A **89**, amphimelibioside B **90**, amphimelibioside C **91**, amphimelibioside D **92**, amphimelibioside E **93**, and amphimelibioside F **94** [19], as shown in Figure 22, are new ceramide dihexosides and were isolated from *Amphimedon* sp. collected near Fukuoka, Japan.

**Figure 23:**
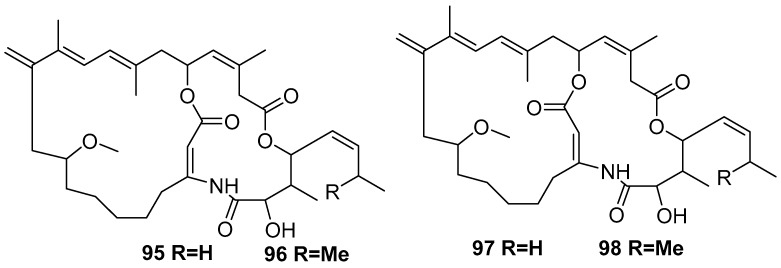
The chemical structures of the compounds (**95**–**98**).

Isolated macrocyclic lactone/lactams are amphilactam A–D **95**–**98** [18], as shown in Figure 23. These four compounds were found to be active against nematodes in vitro with LD_99_ activities of 7.5, 47, 8.5, and 0.39 µg/mL, respectively.

**Figure 24:**
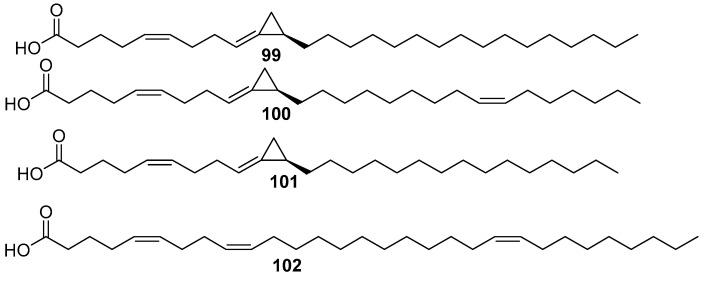
The chemical structures of the compounds (**99**–**102**).

Amphimic acid A, B **99**, **100** displayed inhibitory activity against DNA to poisomerase I with IC_50_ values of 0.47 and 3.2 µM, respectively Amphimic acid C **102** and 5Z,9Z,21Z-triacontatrienoic acid were isolated from the *Amphimedon* sp. **103** [59], as shown in Figure 24.

**Figure 25:**
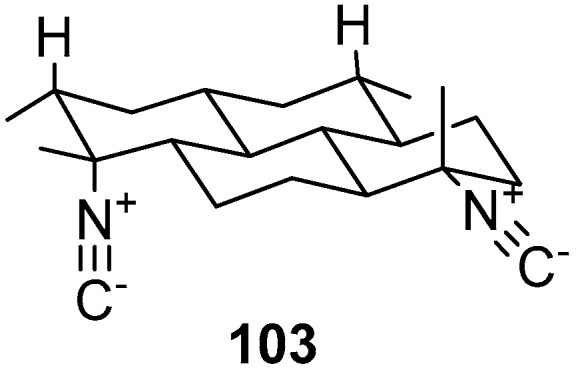
The chemical structure of the compound (**103**).

The terpene diisocyanoadociane **103** [60], as shown in Figure 25, is an example of a compound from another substance class isolated from *Amphimedon* sp.

## 7. Bacteria Associated with the Genus *Amphimedon*

Marine sponges contain a huge diversity of fatty acids which are hypothesized to be composed from bacterial origin. Regarding *Amphimedon terpenensis*, the brominated long-chain fatty acids **29** and **30**, were more distributed in the sponge and its associated bacterial symbionts [61]. However, the role of these fatty acids in marine sponges is unclear, but they may be involved in the relationship between the sponge and their associated bacteria and in protecting the bacterial symbionts from sponge phagocytosis. Amphitoxin [62] is a pyridinium alkaloid isolated from *Amphimedon chloros*, which was previously described as *Amphimedon viridis*. The amphitoxin exhibited broad-spectrum activity against bacteria derived from seawater. Furthermore, the sponge-associated bacteria alpha proteobacteria were resistant against this compound, as well as six different bacterial strains associated with the sponge *A. viridis* [63]. This inhibition may explain the ability of the bacteria to live in close association with their host sponge while having a chemical defense against other microbial pathogens. Moreover, actinomycetes such as *Kocuria*, *Microbacterium*, and *Micrococcus* were cultivated from the *Amphimedon* sp. that had been collected offshore of the Red Sea, Egypt, but were never chemically investigated [64].

Several types of mycobacteria, including a strain closely related to *M. tuberculosis*, have been isolated from *Amphimedon queenslandica*. The strain *Salinispora arenicola* was also isolated from *Amphimedon queenslandica*. This strain can synthesize antimycobacterial compounds [65].

As the polyketide class is well-known for their antibiotic activities, the polyketide synthetase genes of the sponge-derived mycobacteria were examined [66] and found to catalyze the synthesis of mycobacterial outer membrane lipids.

The diversity in the regions in which *Amphimedon* and its different species were collected, play a role in the production of manzamine alkaloids **30**–**50**. They are widely believed to be produced by a symbiotic relationship between the sponge and common or closely related microorganism(s), which may account for the generation of the manzamine alkaloids [67]. Furthermore, there is evidence that sponge-associated microbes have a significant role in the bioconversion of manzamines to the growing number of alkaloids found in sponges producing manzamines and is provided by the biotransformation of 8-hydroxymanzamine A and its enantiomer to manzamine A and ent-12,34-oxamazamine F, respectively [41].

Classes of chemical compounds, such as fatty acids, were used as markers for the the chemodiversity in the genus *Amphimedon*. Several unique fatty acids were isolated from this genus as 17, 18, and 19-hexacosenoic acids. Commonly, there were two biosynthetic pathways that were reported in marine sponges such as 18: ln-7 to 26: ln-7, and the 18: ln-9 to 26: ln-9 routes. While the 18: ln-8 to 26: ln-8 pathway has only recently existed in these invertebrates [28,68] the long chain monounsaturated fatty acids were rare in nature due to their existence in small amounts, especially in microgram quantities [28]. The biosynthesis of the long chain brominated fatty acids in *Amphimedon terpenensis* has previously been explained in past literature. The substituent of bromine was introduced following chain elongation and desaturation of a 16:0 precursor. The formation of 24:2 brominated and 26:2 brominated acids might have been predicted, however, the isolation of a 25:2 brominated acid suggests that sponges may additionally have the capacity to extend chains, desaturate, and functionalize an odd carbon chain length fatty acid. Odd carbon chain fatty acids, such as 15:0 and 17:0, generally have a branched structure rather than straight chain structure which were usually found in bacteria. *Amphimedon terpenensis* contains both cyanobacterial and bacterial symbionts and thus an additional consideration is which of the three cell types, sponge, bacteria, or cyanobacteria contains the brominated fatty acids [69].

Terpenes biosynthesis is under investigation as a rearranged diterpenoid diisocyanoadociane and was isolated from the *Amphimedon* sp. that was collected at the Great Barrier Reef in Australia [60]. Terpenes biosynthesis could be explained by the sponge-associated microorganisms which enable the enzymes to synthesize isoprenoids or alternatively, from the modification in a sponge mediated cell of a symbiont-produced precursor such as isoagelaxanthin [70]. Cyanide incorporation by the *Amphimedon* sp. was first established by Karuso and Scheuer [71] and demonstrated that these sponges utilize carbon and nitrogen from the cyanide precursor. The role of the symbionts may be incorporated in the biosynthesis of isonitriles as the production of diisocyanoadociane from *Amphimedon* sp. in high rates is due to the cyanide production by the symbionts utilization in the sponges or, a metabolic process which can proceed without symbionts.

An important class of metabolites that were reported from the genus *Amphimedon* are manzamine alkaloids owing to their fascinating biological activities. Manzamine alkaloids were biogenetically synthesized from the condensation of ircinals A or B from the *icrinia* sponge [48]. Keramaphidin B, isolated from *Amphimedon* sp., might be the biogenetic precursor of the ircinals and may be produced by hydrolysis of the N2/C3 bond of the 2, 3-imino form of keramaphidin B. In the continuous search for biogenetic precursors of manzamine alkaloids keramaphidin C and keramamine C might be plausible biogenetic precursors of manzamine C, especially considering that keramaphidin C is an important intermediate in the biogenetic pathway of manzamine C generation.

Several *Amphimedon* metabolites were targets for chemical synthesis such as nakadomarin A, owing to its interesting biological applications including antibacterial, cytotoxic and antimicrobial properties [72]. Synthesis was carried out by using a thioamide based strategy [73]. Marine diterpenoid diisocyanoadociane is another example that was synthesized by using intramolecular Michael reaction due to its potent antimalarial activity [74].

## 8. Conclusions

**Figure 26:**
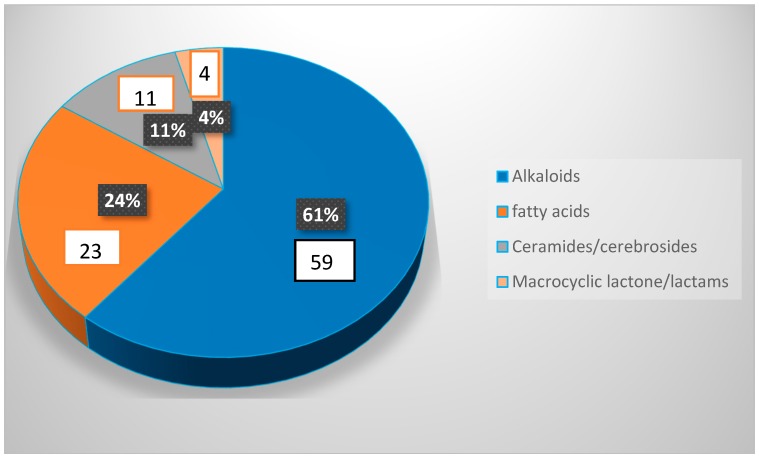
The distribution of different chemical classes of compounds isolated from the genus *Amphimedon*.

**Figure 27:**
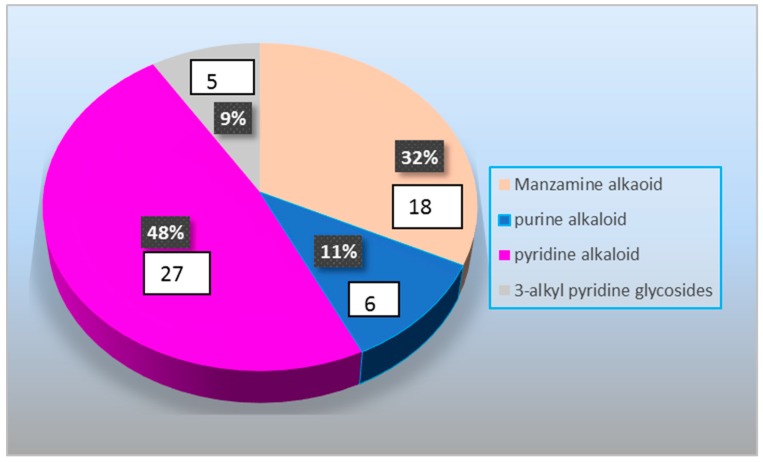
The distribution of different chemical subclasses of the alkaloids compounds isolated from the genus *Amphimedon*.

Marine sponges contain treasures of undiscovered natural metabolites by having a wide spectrum of pharmacological activities. Among the several *Amphimedon* species discovered, *A. viridis*, *A. compressa*, *A. complanata*, and *A. terpenensis* are the richest producers of natural products with various pharmaceutically relevant bioactivities. The research on this genus is interesting and has made variable progression over the years. However, in recent years there is increasing interest in studying sponge-associated microorganisms in order to explain this complex relationship and in order to reveal the facts of whether metabolites originate mainly from the sponge or from their associated microorganism(s). One hundred and four natural products from various marine sponges belonging to the genus *Amphimedon* were reported in literature as well as in the Marinlit databases until 2018. Undescribed species of the sponge represent the highest source of secondary metabolites and thus, the discovery of new species of this sponge indicate that there are still possibilities in isolating new secondary metabolites with predicted strong pharmacological activities. The genus *Amphimedon* and its associated microorganism(s) showed their potential to produce great diversity of chemical leads from the different chemical classes. Alkaloids showed the highest distribution among the different chemical classes followed by fatty acids and then ceramides and cerebrosoides and finally, the lowest percent were the macrocyclic lactones/lactams, as shown in Figure 26. Among the various types of alkaloids pyridine type alkaloids and manzamine alkaloids were the highest followed by purine alkaloids and finally 3-alkyl pyridine glycosides, as shown in Figure 27. These chemical leads exhibited a huge diversity of bioactivities such as antibacterial, antifungal, and anticancer activities.

*Amphimedon* and its associated microorganisms have a broad range of pharmacological activities and, therefore, we must carry out further investigations for the genus and their associated microorganisms as they play an important role in the discovery of new natural products.

## References

[1] Hu G.-P., Yuan J., Sun L., She Z.-G., Wu J.-H., Lan X.-J., Zhu X., Lin Y.-C., Chen S.-P. (2011). Statistical research on marine natural products based on data obtained between 1985 and 2008. Mar. Drugs.

[2] Blunt J.W., Copp B.R., Keyzers R.A., Munro M.H., Prinsep M.R. (2013). Marine natural products. Nat. Prod. Rep..

[3] Blunt J.W., Copp B., Keyzers R.A., Munro M.H., Prinsep M.R. (2014). Marine natural products. Nat. Prod. Rep..

[4] Blunt J.W., Copp B.R., Keyzers R.A., Munro M.H., Prinsep M.R. (2012). Marine natural products. Nat. Prod. Rep..

[5] Blunt J.W., Copp B.R., Keyzers R.A., Munro M.H., Prinsep M.R. (2016). Marine natural products. Nat. Prod. Rep..

[6] Blunt J.W., Copp B.R., Keyzers R.A., Munro M.H.G., Prinsep M.R. (2017). Marine natural products. Nat. Prod. Rep..

[7] Blunt J.W., Carroll A.R., Copp B.R., Davis R.A., Keyzers R.A., Prinsep M.R. (2018). Marine natural products. Nat. Prod. Rep..

[8] Hill R.T. (2004). Microbes from marine sponges: A treasure trove of biodiversity for natural products discovery. Microbial Diversity and Bioprospecting.

[9] Webster N.S., Taylor M.W. (2012). Marine sponges and their microbial symbionts: Love and other relationships. Environ. Microbiol..

[10] Mohamed N.M., Rao V., Hamann M.T., Kelly M., Hill R.T. (2008). Monitoring bacterial diversity of the marine sponge Ircinia strobilina upon transfer into aquaculture. Appl. Environ. Microbiol..

[11] Shieh W.Y., Lin Y.M. (1994). Association of heterotrophic nitrogen-fixing bacteria with a marine sponge of *Halichondria* sp.. Bull. Mar. Sci..

[12] Osinga R., Armstrong E., Burgess J.G., Hoffmann F., Reitner J., Schumann-Kindel G. (2001). Sponge–microbe associations and their importance for sponge bioprocess engineering. Hydrobiologia.

[13] Kubota T., Kamijyo Y., Takahashi-Nakaguchi A., Fromont J., Gonoi T., Kobayashi J.I. (2013). Zamamiphidin A, a new manzamine related alkaloid from an Okinawan marine sponge *Amphimedon* sp.. Org. Lett..

[14] Tsuda M., Kawasaki N., Kobayashi J.I. (1994). Ircinols A and B, first antipodes of manzamine-related alkaloids from an Okinawan marine sponge. Tetrahedron.

[15] Sakurada T., Gill M.B., Frausto S., Copits B., Noguchi K., Shimamoto K., Swanson G.T., Sakai R. (2010). Novel *N*-methylated 8-oxoisoguanines from Pacific sponges with diverse neuroactivities. J. Med. Chem..

[16] Hirano K., Kubota T., Tsuda M., Mikami Y., Kobayashi J.I. (2000). Pyrinodemins B–D, potent cytotoxic bis-pyridine alkaloids from marine sponge *Amphimedon* sp.. Chem. Pharm. Bull..

[17] Takekawa Y., Matsunaga S., van Soest R.W., Fusetani N. (2006). Amphimedosides, 3-alkylpyridine glycosides from a marine sponge *Amphimedon* sp.. J. Nat. Prod..

[18] Ovenden S.P., Capon R.J., Lacey E., Gill J.H., Friedel T., Wadsworth D. (1999). Amphilactams A–D: Novel Nematocides from Southern Australian Marine Sponges of the Genus *Amphimedon*. J. Org. Chem..

[19] Emura C., Higuchi R., Miyamoto T., Van Soest R.W. (2005). Amphimelibiosides A–F, Six New Ceramide Dihexosides Isolated from a Japanese Marine Sponge *Amphimedon* sp.. J. Org. Chem..

[20] Nemoto T., Ojika M., Sakagami Y. (1997). Amphimic acids, novel unsaturated C28 fatty acids as DNA topoisomerase I inhibitors from an Australian sponge *Amphimedon* sp.. Tetrahedron Lett..

[21] Tsuda M., Inaba K., Kawasaki N., Honma K., Kobayashi J.I. (1996). Chiral resolution of (±)-keramaphidin B and isolation of manzamine L, a new β-carboline alkaloid from a sponge *Amphimedon* sp.. Tetrahedron.

[22] Kubota T., Nakamura K., Kurimoto S.-I., Sakai K., Fromont J., Gonoi T., Kobayashi J.I. (2017). Zamamidine D, a manzamine alkaloid from an Okinawan *Amphimedon* sp. marine sponge. J. Nat. Prod..

[23] Yamada M., Takahashi Y., Kubota T., Fromont J., Ishiyama A., Otoguro K., Yamada H., Ōmura S., Kobayashi J.I. (2009). Corrigendum to Zamamidine C, 3,4-dihydro-6-hydroxy-10,11-epoxymanzamine A, and 3,4-dihydromanzamine J *N*-oxide, new manzamine alkaloids from sponge *Amphimedon* sp.. Tetrahedron.

[24] Tsukamoto S., Takahashi M., Matsunaga S., Fusetani N., Van Soest R.W. (2000). Hachijodines A–G: Seven New Cytotoxic 3-Alkylpyridine Alkaloids from Two Marine Sponges of the Genera *Xestospongia* and *Amphimedon*. J. Nat. Prod..

[25] Xu N.J., Sun X., Yan X.J. (2007). A new cyclostellettamine from sponge *Amphimedon compressa*. Chin. Chem. Lett..

[26] Albrizio S., Ciminiello P., Fattorusso E., Magno S., Pawlik J.R. (1995). Amphitoxin, a new high molecular weight antifeedant pyridinium salt from the Caribbean sponge *Amphimedon compressa*. J. Nat. Prod..

[27] Thompson M.N., Gallimore W.A., Townsend M.M., Chambers N.A., Williams L.A. (2010). Bioactivity of amphitoxin, the major constituent of the Jamaican sponge *Amphimedon compressa*. Chem. Biodivers..

[28] Carballeira N.M., Negrón V., Reyes E.D. (1992). Novel monounsaturated fatty acids from the sponges *Amphimedon compressa* and *Mycale laevis*. J. Nat. Prod..

[29] Carballeira N.M., Lopez M.R. (1989). On the Isolation of 2-Hydroxydocosanoic and 2-Hydroxytricosanoic Acids from the marine Sponge *Amphimedon compressa*. Lipids.

[30] Carballeira N.M., Colón R., Emiliano A. (1998). Identification of 2-methoxyhexadecanoic acid in *Amphimedon compressa*. J. Nat. Prod..

[31] Costantino V., Fattorusso E., Imperatore C., Mangoni A., Teta R. (2009). Amphiceramide A and B, novel glycosphingolipids from the marine sponge *Amphimedon compressa*. Eur. J. Org. Chem..

[32] Mitchell S.S., Whitehill A.B., Trapido-Rosenthal H.G., Ireland C.M. (1997). Isolation and characterization of 1,3-dimethylisoguanine from the Bermudian sponge *Amphimedon viridis*. J. Nat. Prod..

[33] Chehade C.C., Dias R.L., Berlinck R.G., Ferreira A.G., Costa L.V., Rangel M., Malpezzi E.L., de Freitas J.C., Hajdu E. (1997). 1,3-Dimethylisoguanine, a new purine from the marine sponge *Amphimedon viridis*. J. Nat. Prod..

[34] Sharangi A.B. (2009). Medicinal and therapeutic potentialities of tea (*Camellia sinensis* L.)—A review. Food Res. Int..

[35] Hirsch S., Kashman Y. (1989). New glycosphingolipids from marine organisms. Tetrahedron.

[36] Carballeira N.M., Restituyo J. (1991). Identification of the new 11,15-icosadienoic acid and related acids in the sponge *Amphimedon complanata*. J. Nat. Prod..

[37] Carballeira N.M., Alicea J. (2001). The first naturally occurring α-methoxylated branched-chain fatty acids from the phospholipids of *Amphimedon complanata*. Lipids.

[38] Sakai R., Higa T., Jefford C.W., Bernardinelli G. (1986). Manzamine A, a novel antitumor alkaloid from a sponge. J. Am. Chem. Soc..

[39] Sakai R., Kohmoto S., Higa T., Jefford C.W., Bernardinelli G. (1987). Manzamine B and C, two novel alkaloids from the sponge *Haliclona* sp.. Tetrahedron Lett..

[40] Kondo K., Shigemori H., Kikuchi Y., Ishibashi M., Sasaki T., Kobayashi J. (1992). Ircinals A and B from the Okinawan marine sponge *Ircinia* sp.: Plausible biogenetic precursors of manzamine alkaloids. J. Org. Chem..

[41] Radwan M., Hanora A., Khalifa S., Abou-El-Ela S.H. (2012). Manzamines: A potential for novel cures. Cell Cycle.

[42] El Sayed K.A., Dunbar D.C., Perry T.L., Wilkins S.P., Hamann M.T., Greenplate J.T., Wideman M.A. (1997). Marine natural products as prototype insecticidal agents. J. Agric. Food Chem..

[43] Kobayashi J.I., Tsuda M., Kawasaki N., Sasaki T., Mikami Y. (1994). 6-Hydroxymanzamine A and 3,4-dihydromanzamine A, new alkaloids from the Okinawan marine sponge *Amphimedon* sp.. J. Nat. Prod..

[44] Mayer A., Gunasekera S.P., Pomponi S.A., Sennett S.H. (2002). Anti-Inflammatory Uses of Manzamines. U.S. Patent.

[45] Takahashi Y., Kubota T., Fromont J., Kobayashi J.I. (2008). Zamamidines A and B, new manzamine alkaloids from the sponge Amphimedon species. Org. Lett..

[46] Yamada M., Takahashi Y., Kubota T., Fromont J., Ishiyama A., Otoguro K., Yamada H., Ōmura S., Kobayashi J.I. (2009). Zamamidine C, 3,4-dihydro-6-hydroxy-10,11-epoxymanzamine A, and 3,4-dihydromanzamine J *N*-oxide, new manzamine alkaloids from sponge *Amphimedon* sp.. Tetrahedron.

[47] Tsuda M., Kawasaki N., Kobayashi J. i. (1994). Keramaphidin C and keramamine C plausible biogenetic precursors of manzamine C from an Okinawan marine sponge. Tetrahedron Lett..

[48] Kobayashi J.I., Tsuda M., Kawasaki N., Matsumoto K., Adachi T. (1994). Keramaphidin B, a novel pentacyclic alkaloid from a marine sponge *Amphimedon* sp.: A plausible biogenetic precursor of manzamine alkaloids. Tetrahedron Lett..

[49] Watanabe D., Tsuda M., Kobayashi J.I. (1998). Three new manzamine congeners from *Amphimedon sponge*. J. Nat. Prod..

[50] Kobayashi J.I., Watanabe D., Kawasaki N., Tsuda M. (1997). Nakadomarin A, a novel hexacyclic manzamine-related alkaloid from *Amphimedon sponge*. J. Org. Chem..

[51] Tsuda M., Hirano K., Kubota T., Kobayashi J.I. (1999). Pyrinodemin A, a cytotoxic pyridine alkaloid with an isoxazolidine moiety from sponge *Amphimedon* sp.. Tetrahedron Lett..

[52] Kura K.I., Kubota T., Fromont J., Kobayashi J.I. (2011). Pyrinodemins E and F, new 3-alkylpyridine alkaloids from sponge *Amphimedon* sp.. Bioorg. Med. Chem. Lett..

[53] Kubota T., Kura K.I., Fromont J., Kobayashi J.I. (2013). Pyrinodemins G–I, new bis-3-alkylpyridine alkaloids from a marine sponge *Amphimedon* sp.. Tetrahedron.

[54] Kubota T., Nishi T., Fukushi E., Kawabata J., Fromont J., Kobayashi J. (2007). Nakinadine A, a novel bis-pyridine alkaloid with a β-amino acid moiety from sponge *Amphimedon* sp.. Tetrahedron Lett..

[55] Nishi T., Kubota T., Fromont J., Sasaki T., Kobayashi J.I. (2008). Nakinadines B–F: New pyridine alkaloids with a β-amino acid moiety from sponge *Amphimedon* sp.. Tetrahedron.

[56] Matsunaga S., Miyata Y., van Soest R.W., Fusetani N. (2004). Tetradehydrohalicyclamine A and 22-hydroxyhalicyclamine A, new cytotoxic bis-piperidine alkaloids from a marine sponge *Amphimedon* sp.. J. Nat. Prod..

[57] Schmitz F.J., Agarwal S.K., Gunasekera S.P., Schmidt P.G., Shoolery J.N. (1983). Amphimedine, new aromatic alkaloid from a pacific sponge, *Amphimedon* sp. Carbon connectivity determination from natural abundance carbon-13-carbon-13 coupling constants. J. Am. Chem. Soc..

[58] Campos P.-E., Wolfender J.-L., Queiroz E.F., Marcourt L., Al-Mourabit A., De Voogd N., Illien B., Gauvin-Bialecki A. (2017). Amphimedonoic acid and psammaplysene E, novel brominated alkaloids from *Amphimedon* sp.. Tetrahedron Lett..

[59] Nemoto T., Yoshino G., Ojika M., Sakagami Y. (1997). Amphimic acids and related long-chain fatty acids as DNA topoisomerase I inhibitors from an Australian sponge, *Amphimedon* sp.: Isolation, structure, synthesis, and biological evaluation. Tetrahedron.

[60] Garson M.J., Thompson J.E., Larsen R.M., Battershill C.N., Murphy P.T., Bergquist P.R. (1992). Terpenes in sponge cell membranes: Cell separation and membrane fractionation studies with the tropical marine sponge *Amphimedon* sp.. Lipids.

[61] Sathiyanarayanan G., Saibaba G., Kiran G.S., Yang Y.-H., Selvin J. (2017). Marine sponge-associated bacteria as a potential source for polyhydroxyalkanoates. Crit. Rev. Microbiol..

[62] Kelman D., Kashman Y., Hill R.T., Rosenberg E., Loya Y. (2009). Chemical warfare in the sea: The search for antibiotics from Red Sea corals and sponges. Pure Appl. Chem..

[63] Kelman D., Kashman Y., Rosenberg E., Ilan M., Ifrach I., Loya Y. (2001). Antimicrobial activity of the reef sponge Amphimedon viridis from the Red Sea: Evidence for selective toxicity. Aquat. Microb. Ecol..

[64] Abdelmohsen U.R., Bayer K., Hentschel U. (2014). Diversity, abundance and natural products of marine sponge-associated actinomycetes. Nat. Prod. Rep..

[65] Izumi H., Gauthier M.E., Degnan B.M., Ng Y.K., Hewavitharana A.K., Shaw P.N., Fuerst J.A. (2010). Diversity of Mycobacterium species from marine sponges and their sensitivity to antagonism by sponge-derived rifamycin-synthesizing actinobacterium in the genus Salinispora. FEMS Microbiol. Lett..

[66] Walsh C.T. (2004). Polyketide and nonribosomal peptide antibiotics: Modularity and versatility. Science.

[67] Hu J.F., Hamann M.T., Hill R., Kelly M. (2003). The manzamine alkaloids. Alkaloids Chem. Biol..

[68] Carballeira N.M., Shalabi F., Maldonado M.E. (1990). Identification of the new 18-hexacosenoic acid in the sponge *Thalysias juniperina*. Lipids.

[69] Garson M.J., Zimmermann M.P., Hoberg M., Larsen R.M., Battershill C.N., Murphy P.T. (1993). Isolation of brominated long-chain fatty acids from the phospholipids of the tropical marine sponge *Amphimedon terpenensis*. Lipids.

[70] Garson M.J., Partali V., Liaaen-Jensen S., Stoilov I.L. (1988). Isoprenoid biosynthesis in a marine sponge of the Amphimedon genus: Incorporation studies with [1-^14^C] acetate, [4-^14^C] cholesterol and [2-^14^C] mevalonate. Comp. Biochem. Physiol. B.

[71] Karuso P., Scheuer P.J. (1989). Biosynthesis of isocyanoterpenes in sponges. J. Org. Chem..

[72] Young I.S., Kerr M.A. (2007). Total synthesis of (+)-nakadomarin A. J. Am.Chem. Soc..

[73] Chavda J.K., Procopiou P.A., Horton P.N., Coles S.J., Porter M.J. (2014). Synthetic Studies Towards the Core Structure of Nakadomarin A by a Thioamide-Based Strategy. Eur. J. Org. Chem..

[74] Fairweather K.A., Crabtree S.R., Mander L.N. (2008). A formal total synthesis of the marine diterpenoid, diisocyanoadociane. Strategies and Tactics in Organic Synthesis.

